# Proteomic analysis of the influence of Cu^2+^ on the crystal protein production of *Bacillus thuringiensis* X022

**DOI:** 10.1186/s12934-015-0339-9

**Published:** 2015-10-05

**Authors:** Xuemei Liu, Mingxing Zuo, Ting Wang, Yunjun Sun, Shuang Liu, Shengbiao Hu, Hao He, Qi Yang, Jie Rang, Meifang Quan, Liqiu Xia, Xuezhi Ding

**Affiliations:** College of Life Science, Hunan Provincial Key Laboratory of Microbial Molecular Biology-State Key Laboratory Breeding Base of Microbial Molecular Biology, Hunan Normal University, Changsha, 410081 China

**Keywords:** *Bacillus thuringiensis*, Cu^2+^, Insecticidal crystal proteins, Proteome, PhaR

## Abstract

**Background:**

*Bacillus thuringiensis* X022, a novel strain isolated from soil in China, produces diamond-shaped parasporal crystals. Specific mineral nutrients, such as Mg, Cu, and Mn, influence insecticidal crystal proteins (ICP) expression and the effects of these elements vary significantly. However, the molecular mechanisms of the effects caused by mineral elements have yet to be reported.

**Results:**

The ICP are mainly composed of Cry1Ca, Cry1Ac, and Cry1Da, which have molecular weights of about 130 kDa. ICP production was most efficient when Cu^2+^ was added at concentrations ranging from 10^−6^ to 10^−4^ mol/L at an initial pH of 8.0. Addition of Cu^2+^ also evidently increased the toxicity of fermentation broth to *Spodoptera exigua* and *Helicoverpa armigera*. After analyzing changes in proteome and fermentation parameters caused by Cu^2+^ addition, we propose that Cu^2+^ increases PhaR expression and consequently changes the carbon flow. More carbon sources was used to produce intracellular poly-β-hydroxybutyrate (PHB). Increases in PHB as a storage material bring about increases of ICP production.

**Conclusions:**

*Bacillus thuringiensis* X022 mainly expresses Cry1Ca, Cry1Ac, and Cry1Da. Cu^2+^ increases the expression of Cry1Da, Cry1Ca, and also enhances the toxicity of fermentation broth to *S. exigua* and *H. armigera.*

**Electronic supplementary material:**

The online version of this article (doi:10.1186/s12934-015-0339-9) contains supplementary material, which is available to authorized users.

## Background

The toxicity of insecticidal crystal proteins (ICP) produced by *Bacillus thuringiensis* to agricultural pests and disease vectors is well known. The species has been successfully used as a microbial insecticide and provides large sources of genes for recombinant bacteria and insect-resistant transgenic plants [1–3]. Because of the development of insect resistance to *B. thuringiensis* [4, 5], however, screening for new serotypes that bear novel crystal genes is an important endeavor.

ICP are secondary metabolites, and certain conditions are necessary for their efficient synthesis. Optimization of fermentation conditions can lead to high production of ICP. Some metal ions act as cofactors in holoenzymes and influence the activity of enzyme proteins, ultimately changing the metabolic processes of organisms. Reports showed that some metal ions at trace amounts can significantly stimulate or suppress ICP synthesis. Içgen et al. [6] studied the effects of mineral elements, including MgSO_4_·7H_2_O, CaCl_2_·2H_2_O, MnSO_4_·H_2_O, CuSO_4_·5H_2_O, FeCl_3_, and ZnSO_4_·7H_2_O, on the biosynthesis of crystal proteins in *B. thuringiensis* 81. Results showed that Mg and Cu are the most important metals for the biosynthesis of 135 and 65 kDa toxin components, in that the former was essential and the latter was greatly stimulatory at 10^−7^–10^−6^ mol/L concentration. Mn favors toxin production at concentrations ranging from 10^−5^ to 3 × 10^−4^ mol/L, whereas Zn and Ca have no effect on toxin formation. Özkan et al. [7] investigated the factors influencing Cry11Aa and Cry4Ba synthesis in *B. thuringiensis* subsp. *israelensis* HD500. Results showed that Mn is the most critical element for the biosynthesis of both toxins at 10^−6^ mol/L concentration. Mg and Ca favor toxin production at concentrations of 8 × 10^−3^ and 5.5 × 10^−4^ mol/L, respectively, whereas Fe, Zn, and Cu do not favor biosynthesis. Kurt et al. [8] found that omission of FeSO_4_ from the medium results in reductions in Cry3Aa yield in *B. thuringiensis* Mm2 by about 50 %.

From these studies, we can conclude that specific mineral nutrients, such as Mg, Cu, and Mn, influence ICP expression and that the effects of these elements vary significantly according to the strains applied and ICP types produced. However, the molecular mechanisms of the effects caused by mineral elements have not been reported yet.

*Bacillus thuringiensis* X022 was isolated in this study and showed strong insecticidal activity against *Spodoptera exigua* and *Helicoverpa armigera*. Adding an appropriate amount of Cu^2+^ to the medium evidently improved the synthesis of 130 kDa ICP. We performed two-dimensional liquid chromatography-tandem mass spectrometry (2D-LC–MS/MS) to explore the regulatory mechanisms of ICP biosynthesis. This study presents new insights into the effects of metal ions on the metabolic processes and ICP yields of *B. thuringiensis* and provides new data on the regulation of ICP production.

## Results and discussion

### Characterization and identification of the novel strain *Bacillus thuringiensis X022*

**Fig. 1:**
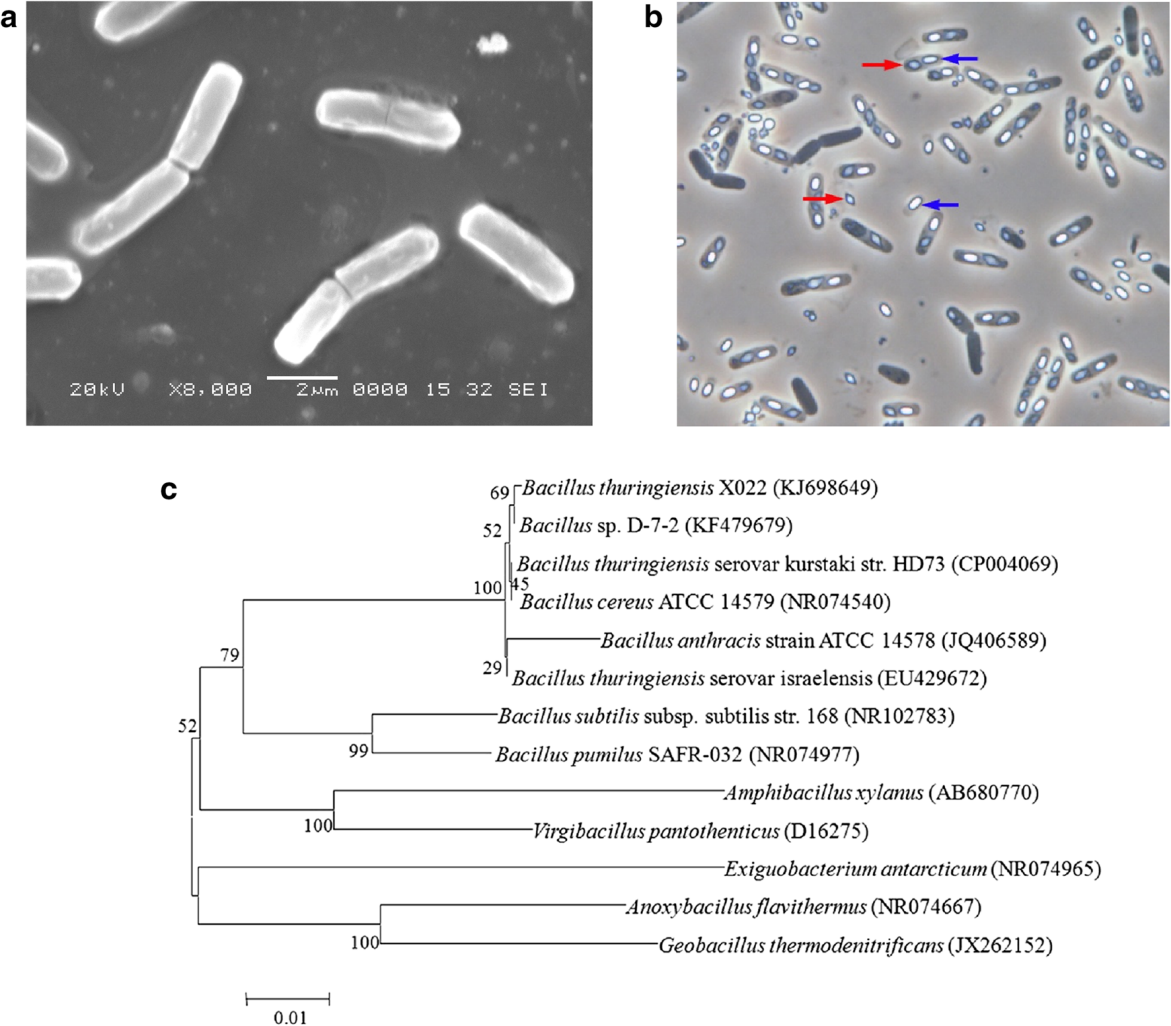
Characterization and identification of *B. thuringiensis* X022. **a** *B. thuringiensis* X022 in the vegetative phase observed through a scanning electron microscope (×8000). **b** *B. thuringiensis* X022 in the spore-release phase observed through phase-contrast microscope (×1000; : *diamond* parasporal crystal, : endospore). **c** Phylogenetic tree constructed based on 16S rRNA gene sequences. The rooted tree was constructed using the Kimura-2-Parameter model and N–J method with bootstrap values calculated from 1000 resamplings. *Numbers at each node* indicate the percentage of bootstrap support. *Letters in brackets* following each bacterial name indicate the 16S rRNA gene sequence accession numbers in GenBank. The *scale bar* represents 0.01 substitutions per site

*Bacillus thuringiensis* strain X022 in the vegetative phase is rod shaped and around 2.5–3.8 μm × 0.5–0.7 μm in size (Fig. 1a). When it enters the sporulation phase, the strain gradually forms spores and diamond-shaped parasporal crystals (Fig. 1b).

The 16S rRNA gene sequence of the strain was uploaded in GenBank of the NCBI database under accession number KJ698649. A phylogenetic tree based on the 16S rRNA gene sequence of the strain was constructed (Fig. 1c). Its 16S rRNA gene is highly homologous to that of *B. thuringiensis* serovar *kurstaki* str. HD73 (99.80 % identity).

### Effects of pH and Cu^2+^ on ICP biosynthesis

**Fig. 2:**
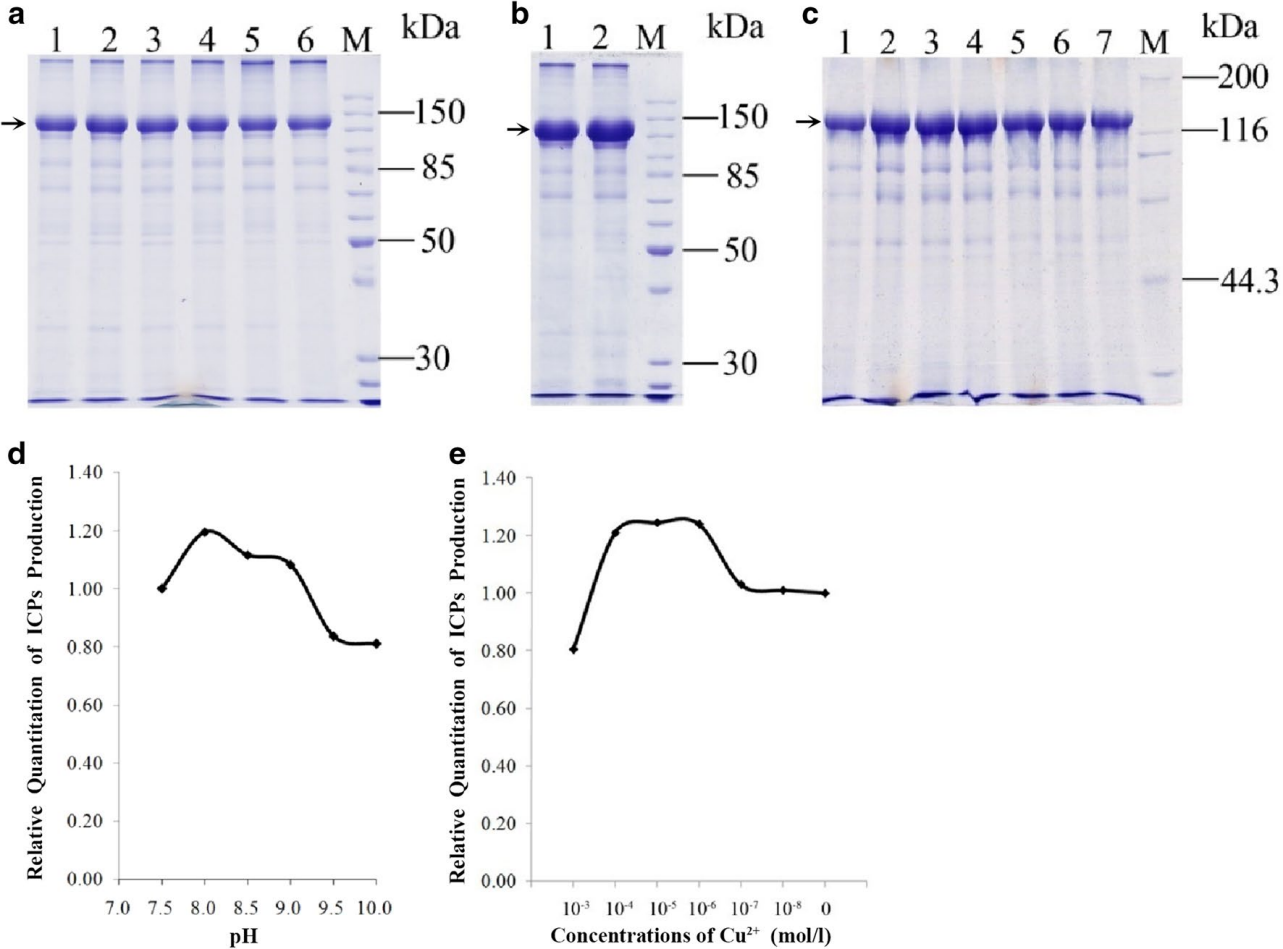
Effect of initial pH and Cu^2+^ on 130 kDa ICP synthesis by *B. thuringiensis* X022 (*arrows* indicate the position of 130 kDa ICP). **a** Effect of initial pH as detected by SDS-PAGE. *Lanes 1*–*6* represent pH 7.5–10.0, *M* protein marker; **b** Effect of 10^−6^ mol/L Cu^2+^ as detected by SDS-PAGE. *Lane 1* without Cu^2+^ added, *lane 2* with 10^−6^ mol/L Cu^2+^ added. **c** Effect of different concentrations of Cu^2+^ as detected by SDS-PAGE. *Lanes* *1*–*6* represent concentrations ranging from 10^−3^ to 10^−8^ mol/L, *lane 7* without Cu^2+^ added, *M* protein marker. **d** Relative quantification of ICP production at different initial pH as analyzed with MiBio software. ICP production at pH 7.5 was set as 1.00. **e** Relative quantification of ICP production at different Cu^2+^ concentrations as analyzed by MiBio software. ICP production in CK (without Cu^2+^) was set as 1.00

The effect of initial pH on ICP biosynthesis was investigated with varying pH ranging from 7.5 to 10.0 (the pH was obtained before sterilization). The relative amount of crystal protein produced was tested by SDS-PAGE. Results showed that 130 kDa ICP production was maximal when the initial pH of the medium was adjusted to 8.0. By contrast, we observed that this isolate doesn’t grow very well at either lower or higher than pH 8.0, and therefore doesn’t produce much ICP (Fig. 2a, d).

**Fig. 3:**
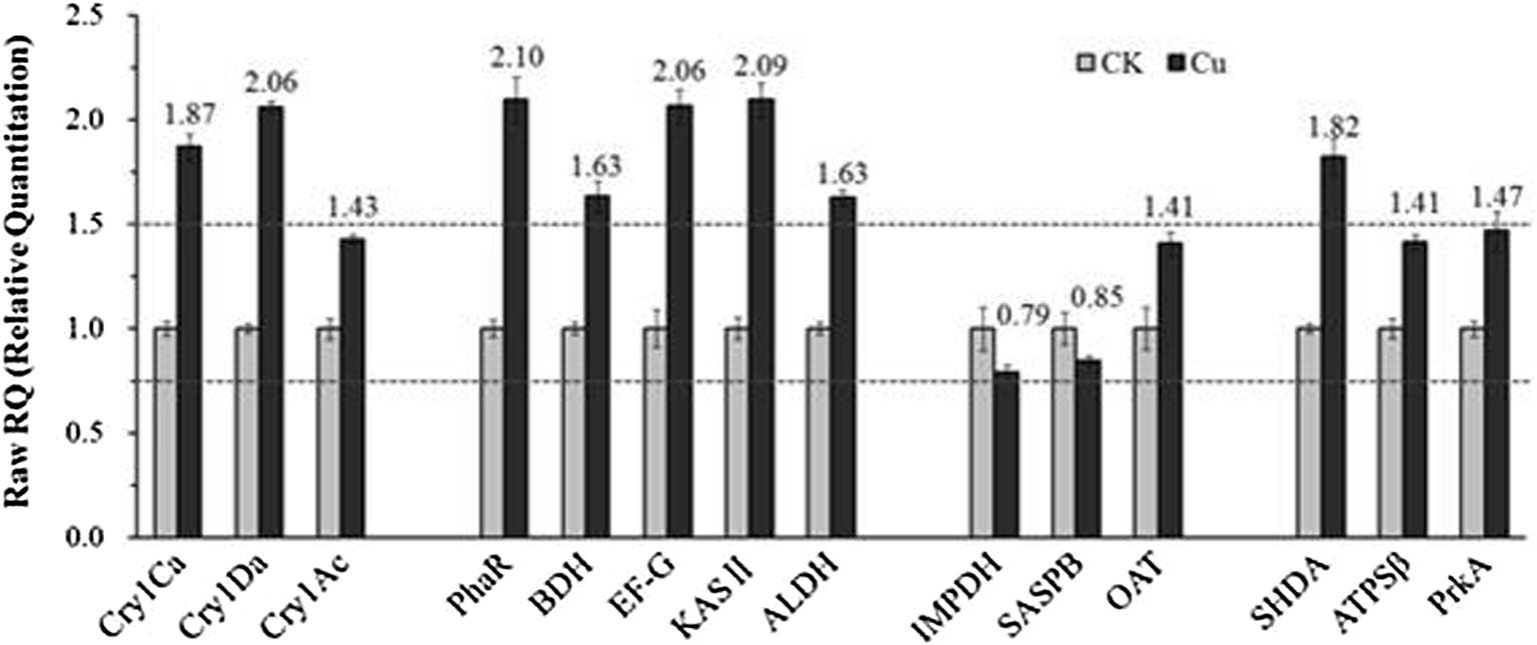
Real-time qRT-PCR analysis of selected genes. qRT-PCR was used to substantiate the differential expression patterns of 14 selected genes (*cry1Ca, cry1Da, cry1Ac, phaR, BDH, EF*-*G, KAS II, ALDH, SHDA, ATPSβ, PrkA, IMPDH, SASPB*, and *OAT*). mRNA levels after 31 h of culture in the original medium (CK) and medium to which Cu^2+^ had been added (Cu) were analyzed as values relative to the 16S rRNA gene. The ratio value for CK was set to 1. *Error bars* are calculated from three independent determinations of mRNA abundance in each sample

**Fig. 4:**
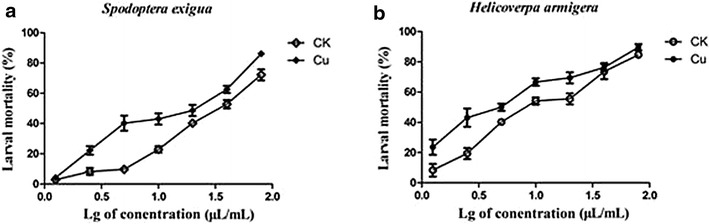
Dose-dependent mortality assays of *B. thuringiensis* X022 toxicity to **a** *S. exigua* and **b** *H. armigera*. CK: fermentation broth from the original medium; Cu: fermentation broth from the medium to which 10^−6^ mol/L Cu^2+^ had been added. Each *point* represents the mean and standard deviation of three independent experiments

SDS-PAGE showed that the production of 130 kDa crystal proteins was increased by 21 % and that the optimal Cu^2+^ concentration ranges from 10^−6^ to 10^−4^ mol/L (Fig. 2c, e). emPAI semi-quantitative analysis of ICP (Additional file 1: Table S3) and qRT-PCR (Fig. 3) showed that Cu^2+^ increases the expression of Cry1Da and Cry1Ca. Toxicity tests demonstrated that Cu^2+^ can increases the toxicity of *B. thuringiensis* X022 to *S. exigua* and *H. armigera* (Fig. 4).

Içgen et al. [6] found that Cu greatly stimulates the biosynthesis of 135 and 65 kDa toxin components in *B. thuringiensis* 81 at concentrations ranging from 10^−7^ to 10^−6^ mol/L. However, Özkan et al. [7] demonstrated that Cu does not favor the biosynthesis of Cry11Aa and Cry4Ba in *B. thuringiensis* subsp. *israelensis* HD500. The effect of Cu^2+^ on ICP biosynthesis may vary according to different strains. The present study showed that appropriate concentrations of Cu^2+^ have positive effects on the expression of Cry1Da and Cry1Ca in *B. thuringiensis* X022; this effect, however, is not significant on Cry1Ac. Oves et al. [9] found that *B. thuringiensis* strain OSM29 shows obvious metal-removing potential; the biosorption capacity of strain OSM29 for Cu is 91.8 % at 25 mg/L initial metal ion concentration. Thus, Cu^2+^ can be absorbed by *B. thuringiensis* and, after entering the cell, bring about changes in metabolic regulation that can affect bacterial growth and ICP synthesis.

### Effects of Cu^2+^ on insecticidal activity

Dose-dependent mortality assays showed that the fermentation broth from the medium with 10^−6^ mol/L Cu^2+^ (Cu) presents higher toxicity to both *S. exigua* and *H. armigera* than the fermentation broth from the original medium (CK) (Fig. 4). The LC_50_ of the original medium (CK) was 1.052 μL/mL at 48 h, and with 10^−6^mol/L Cu^2+^ was 0.484 μL/mL against *S. exigua*; the LC_50_ of the original medium (CK) was 0.518 μL/mL at 48 h, and with 10^−6^ mol/L Cu^2+^ was 0.240 μL/mL against *H. armigera*, which determined by probit analysis demonstrated that Cu^2+^ increases the toxicity of the fermentation broth to *S. exigua* by 1.17 time and to *H. armigera* by 1.16 time (Table 1). The results show that the medium with 10^−6^ mol/L Cu^2+^ has been found to increase the efficacy and potency of *B. thuringiensis* toxins in insect control.

### Changes in growth parameters caused by Cu^2+^

**Fig. 5:**
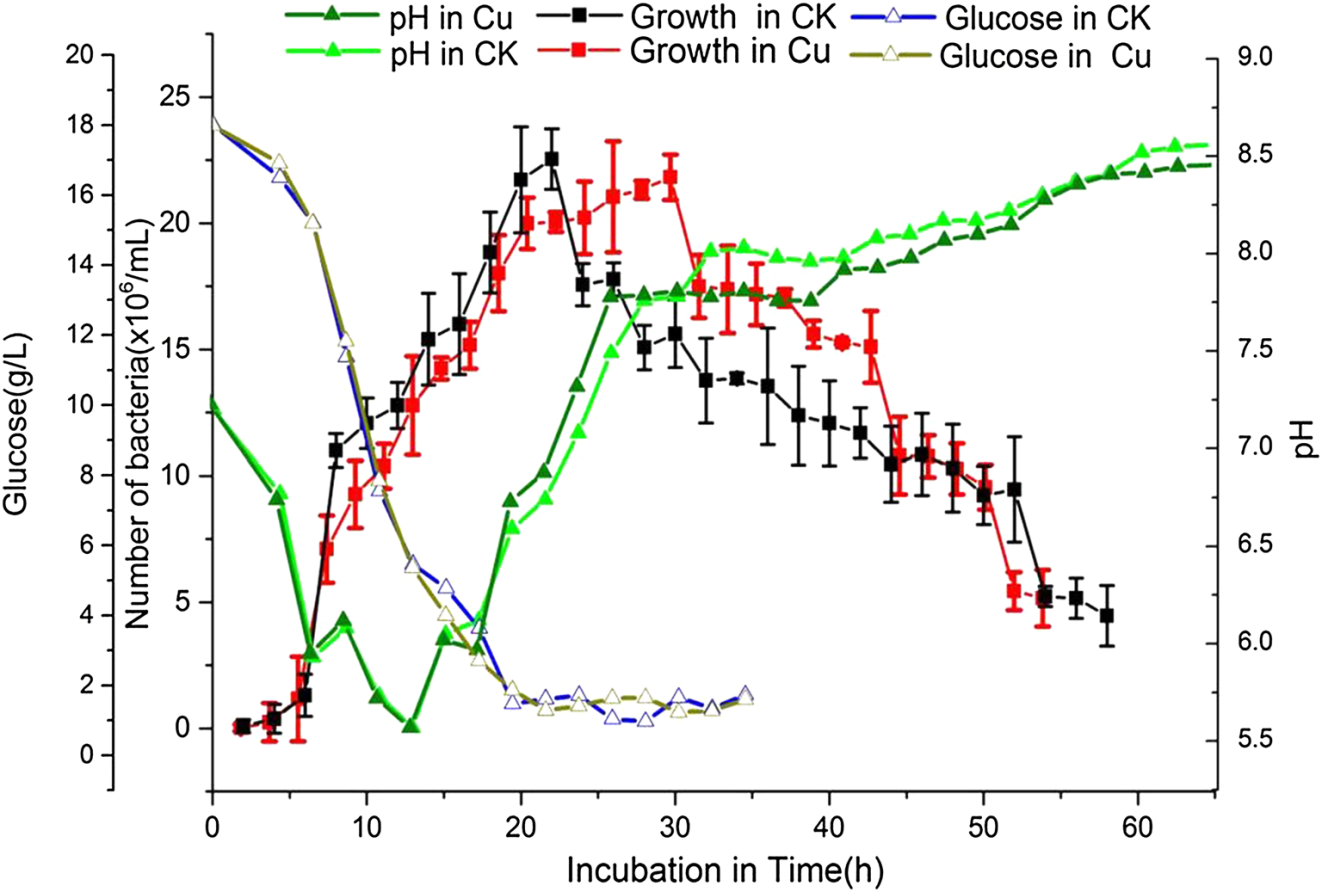
Growth parameters of the *B. thuringiensis* X022. CK represents the original medium without additives, and Cu represents the medium with 10^−6^ mol/L Cu^2+^

**Fig. 6:**
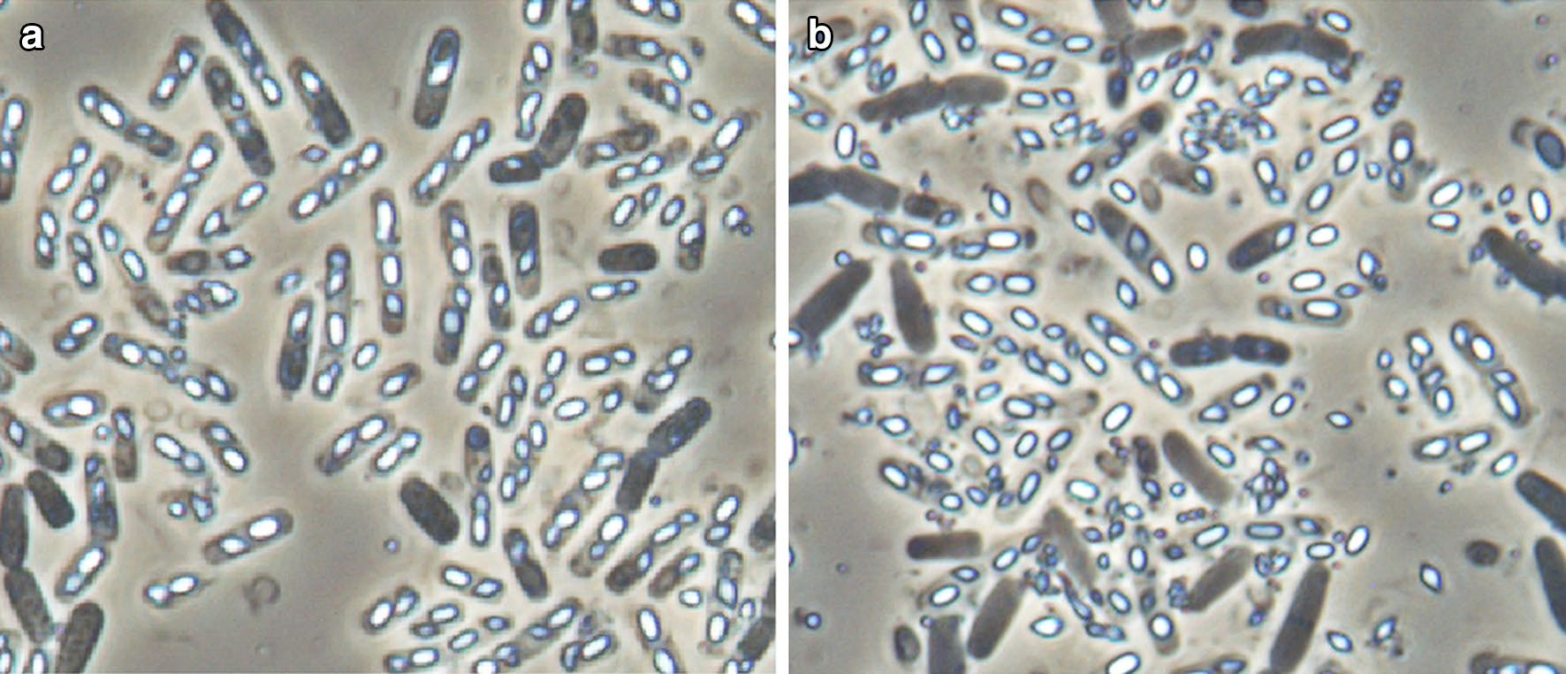
Phase-contrast micrograph of the cell morphology of *B. thuringiensis* X022 (×1000 magnification) after 46 h of cultivation in **a** the original medium and **b** the medium to which 10^−6^ mol/L Cu^2+^ had been added

Cell concentrations, glucose consumption, and pH variation were monitored during bacterial cultivation (Fig. 5). From the growth chart obtained, *B. thuringiensis* X022 appears to grow slowly in the lag phase at 0–4 h. Bacterial growth then enters the logarithmic phase at 4–18 h and then proceeds to the early stage of the stationary phase at 18–28 h. While no differences were observed between CK and Cu, after 28 h of cultivation, the strain declined quickly in CK but remained in the stationary phase for another 8 h in Cu. Compared with the original fermentation medium, the fermentation medium added Cu^2+^ is a clear plateau and from 28 h (the stability mid) the situation has been continued to decline phase. Comparing the bacterial growth curves obtained, we found that Cu^2+^ addition results in a prolonged stationary phase, the cell concentration in Cu is obviously higher than that in CK. We also found that Cu^2+^ addition causes rapid release of spores and crystals (Fig. 6). From the pH variation curves obtained, we discovered that Cu^2+^ brought about rapid pH rebound, prolonged pH plateau phase, and low pH in the plateau phase. No significant difference was observed between the glucose consumption of the two media.

Because crystal proteins are expressed in the stationary phase, prolonging the stationary phase may directly result in increases of ICP synthesis. pH affects nutrient ionization in the medium, influences nutrient absorption, and affects the conversion and utility of nutrients by regulating the activity of various enzymes. Thus, pH finally influences growth, sporulation, and ICP synthesis. If pH does not rebound after the logarithmic phase, *B. thuringiensis* would be unable to form spores and crystals (Rogoff and Yousten [13]).

The effect of air flux on ICP biosynthesis was investigated under rotation speeds varying from 160 to 280 rpm and a culture temperature of 30 °C. The relative amount of crystal protein produced was determined by SDS-PAGE. Results showed a maximum yield of 130 kDa ICP when the rotation speed is adjusted to 200 rpm. The isolate did not appear to grow well at rates either lower or higher than 200 rpm and, therefore, was unable to produce a significant amount of ICP.

The statistical significance of ICP biosynthesis determined by SDS-PAGE using the Gro-gel biomedical image analysis under different conditions, fluxes, pH, and Cu concentration was P < 0.05.

### Proteomic analysis of *B. thuringiensis* X022

Proteins extracted from cells in the spore-release period (44 h) after cultivation in CK and Cu were digested with trypsin, and the resultant peptide mixtures were independently analyzed by 2D-LC–MS/MS. A total of 813 proteins were identified; 651 and 566 proteins were identified in CK and Cu, respectively; and 404 proteins were commonly expressed in both media (Additional file 1: Fig. S1B). The number of proteins in Cu was obviously lower than that in CK. This phenomenon may be explained by several reasons. First, Cu^2+^ may inhibit the expression of some proteins. Second, Cu^2+^ may cause shortening of the fermentation cycle (Fig. 6), early entrance into the decline phase, and faster protein degradation, ultimately resulting in fewer proteins detected in the spore-release period.

### *B. thuringiensis* X022 mainly expresses Cry1Ca, Cry1Ac, and Cry1Da

2D-LC–MS/MS revealed that the novel strain *B. thuringiensis* X022 mainly expresses Cry1Ca, Cry1Ac, and Cry1Da (Additional file 1: Table S2). qRT-PCR analysis using specific primers (Additional file 1: Table S1) further proved the transcription of the three crystal proteins. Three other protoxins (Cry1F, insecticidal protein 2, and Cry1 type crystal protein) were also detected by 2D-LC–MS/MS in Cu medium. Cu^2+^ may activate the expression of specific protoxins. However, as theses protoxins determined had only one unique peptide detected, their expressions require further confirmation.

**Table 1:** LC_50_ analysis of *B. thuringiensis* X022 fermentation broth from two different medium

Medium	LC_50_ (μg/mL) to *S. exigua*	95 % CI	Slope	LC_50_ (μg/mL) to *H. armigera*	95 % CI	Slope
CK^a^	1.052	0.692–2.172	0.085	0.518	0.371–0.864	0.075
Cu^b^	0.484	0.257–0.544	0.073	0.240	0.146–0.441	0.069

Non-overlapping 95 % confidence intervals of LC_50_ were used as the criteria to determine significant difference of toxicities among different formulations of acetamiprid

*CI* confidence interval

^a^CK represent fermentation broth from the original medium

^b^Cu represent fermentation broth from 10^−6^ mol/L Cu^2+^ added medium

### Functional classification

**Fig. 7:**
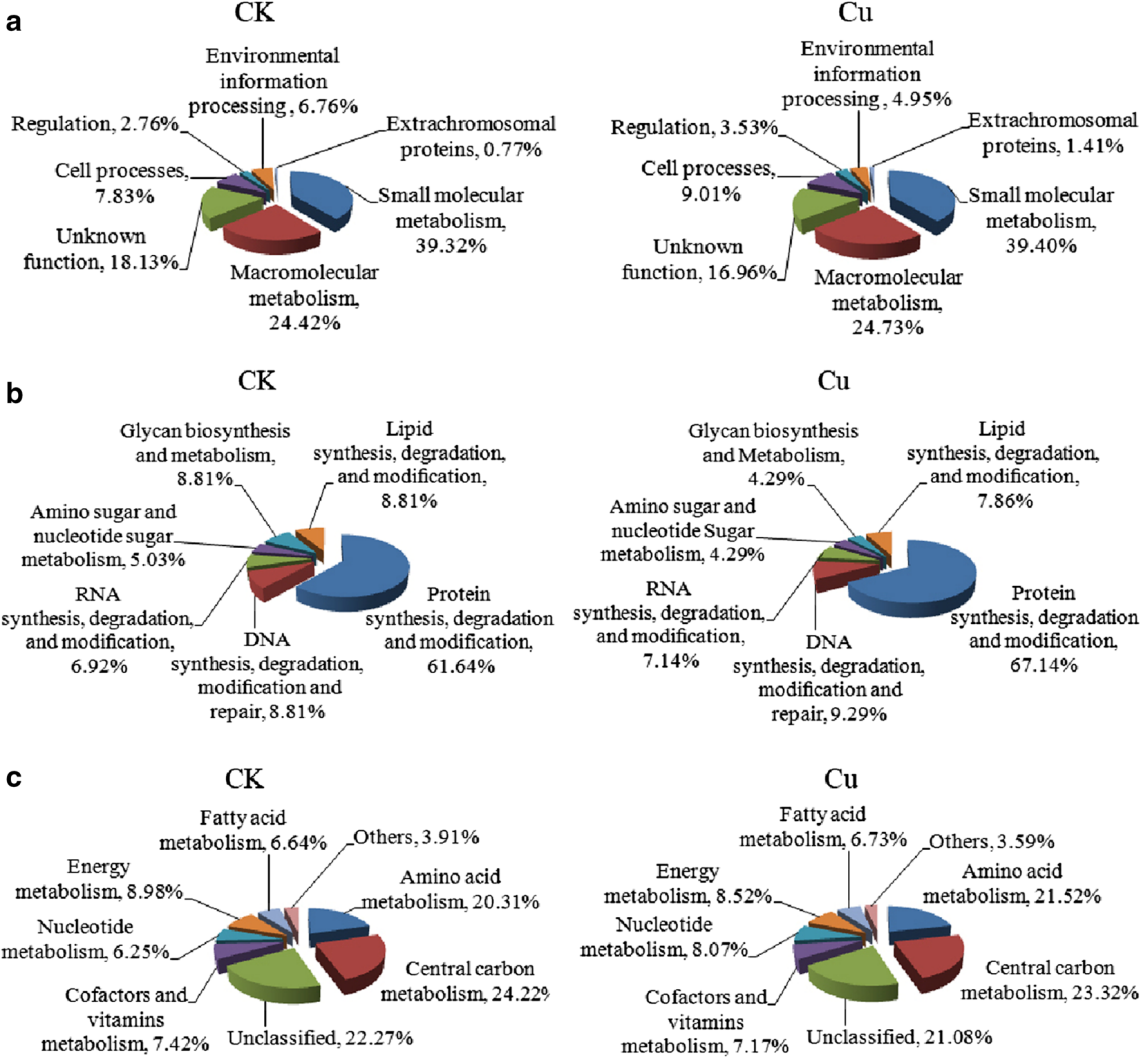
Functional classification of the identified proteins obtained from the spore-release period in *B. thuringiensis* X022. **a** Functional classification of the total identified proteins, **b** subclassification of macromolecular metabolic proteins, and **c** subclassification of identified small molecular metabolic proteins. CK: proteins from the original medium; Cu: proteins from the medium to which 10^−6^ mol/L Cu^2+^ had been added

The proteins were classified into seven functional groups: small molecular metabolism, macromolecular metabolism, cell processes, regulation, environmental information processing proteins, extrachromosomal proteins, and unknown function (Fig. 7a; Additional files 2, 3, 4). Of these groups, the proteins were mainly distributed in small molecular metabolism (CK 39.32 %, Cu 39.40 %) and macromolecular metabolism (CK 24.42 %, Cu 24.73 %). The orders of these categories showed no difference between the two media.

**Fig. 8:**
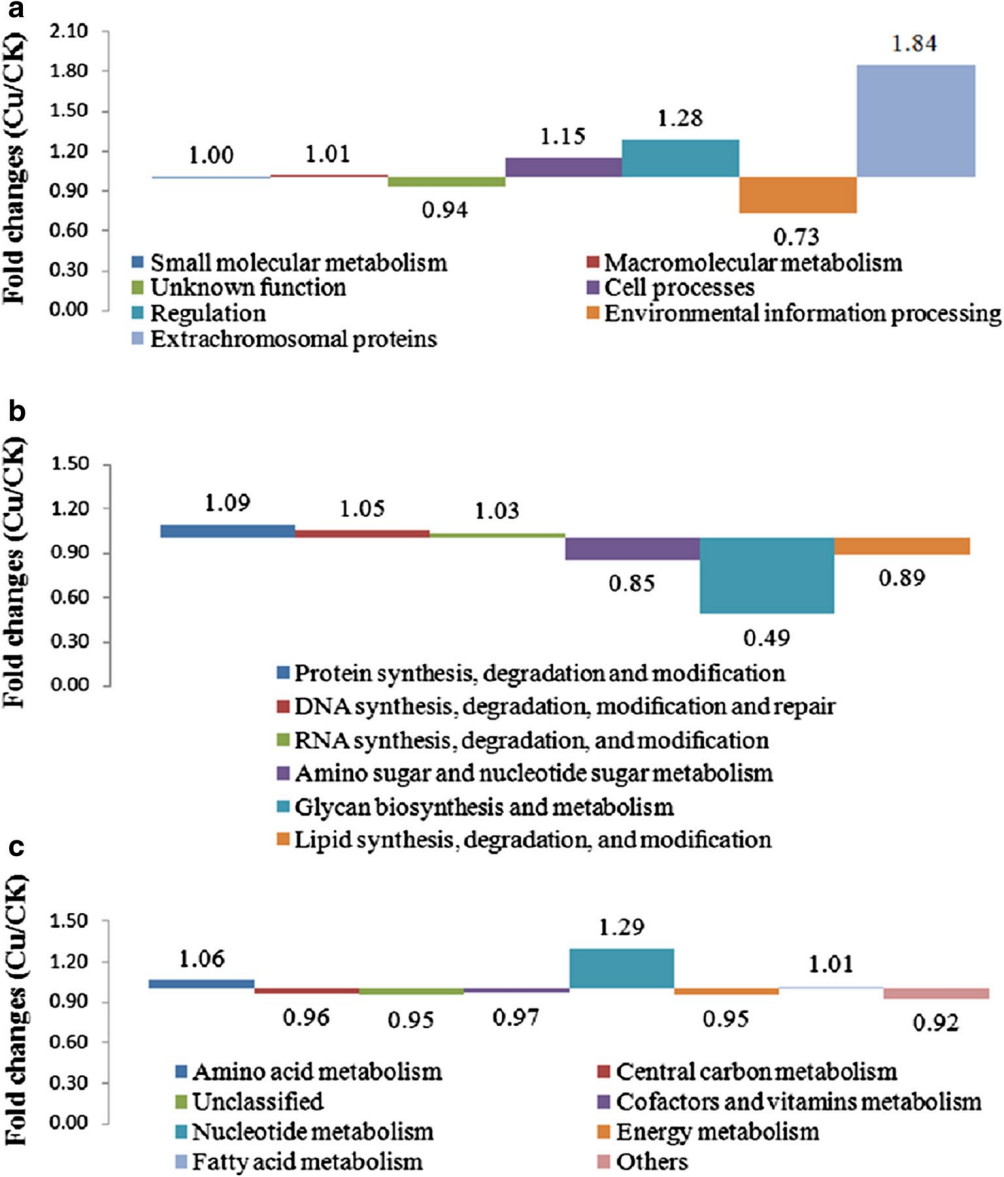
Fold changes of the functional categories of the proteins between Cu and CK. **a** Total identified proteins, **b** macromolecular metabolic proteins, and **c** small molecular metabolic proteins. CK represents proteins from the original medium; Cu represents proteins from the medium to which 10^−6^ mol/L Cu^2+^ had been added

Fold changes of functional categories between Cu and CK were computed (Fig. 8a), and the calculation was done as follows: the percentage of a specific category from Cu was divided by the percentage of that category from CK. A fold-change value >2 indicates a significant increase of a particular category in Cu, whereas a fold-change value <0.5 indicate a significant decrease. Results revealed differences in environmental information-processing proteins and extrachromosomal proteins only. The former which mainly consists of transport proteins such as ABC transporter, decreased, whereas the latter, which is composed of phage-related functions and protoxins, increased.

Huang et al. [10] analyzed the proteome of *B. thuringiensis* subsp. *kurstaki* 4.0718 strain at different growth phases and category of proteins shows the lowest expression in the early sporulation phase, during which the synthesis of ICP was extremely active. Wang et al. [11] found that most genes involved in amino acid transport and biosynthesis are downregulated in the sporulation period, which indicates that the amino acid sources from these two pathways are limited. Thus, that amino acid requirements during sporulation are fulfilled by protein turnover was proposed. Monro [12] proved that 80 % of the amino acids for ICP synthesis come from protein turnover through a radioisotopic tracer experiment. Protein turnover as the major source of amino acids during sporulation can explain why downregulation of environmental information-processing proteins in the Cu^2+^ added medium does not inhibit ICP expression, given that sporulation is a mechanism used by *B. thuringiensis* to resist unfavorable environmental conditions. Reduces the expression of environmental information-processing proteins, and utilizes carbon and nitrogen from intracellular poly-β-hydroxybutyrate (PHB), lipids and proteins stored in the logarithmic phase during sporulation.

The macromolecular and small molecular metabolism proteins were further sorted according to the metabolic phases in which they are involved (Fig. 7b, c; Additional files 2, 3, 4). Fold changes of subcategories were also computed (Fig. 8b, c). For macromolecular metabolism, a large majority of the proteins were involved in protein synthesis, degradation and modification (Fig. 7b; Additional file 1). The subcategory of glycan biosynthesis and metabolism proteins showed downregulation (Fig. 8b), reducing the expression of extracellular polysaccharides. More carbon sources can be used to synthesize intracellular PHB.

Other subcategories showed no significant difference. For small molecular metabolism, proteins that take part in amino acid metabolism and central carbon metabolism comprised the largest subcategory (Fig. 7c; Additional file 1). Fold changes of subcategories in small molecular metabolism showed no significant difference (Fig. 8c).

### emPAI semiquantitative comparative analysis

The abundance of the identified proteins was calculated using emPAI values to investigate their differential expression. Protein abundance was considered significantly up- or downregulated when emPAI ratios between Cu and CK from two replicates were both higher than 1.25 or lower than 0.8. Results showed that Cu^2+^ upregulated 27 types of proteins (Table 2) and downregulated 25 types of proteins (Additional file 1: Table S4). Upregulated proteins included 3-oxoacyl-[acyl-carrier-protein] synthase 2 (KAS II), aldehyde dehydrogenase (ALDH), elongation factor G (EF-G), 3-hydroxybutyrate dehydrogenase (BDH), and PhaR protein. Downregulated proteins included succinate dehydrogenase subunit A (SHDA), ATP synthase subunit beta (ATPSβ), and protein PrkA. For the ICP, emPAI semiquantitative comparative analysis showed that 10^−6^ mol/L Cu^2+^ promotes the synthesis of Cry1Da and Cry1Ca (Additional file 1: Table S3).

**Table 2:** The list of the proteins up-regulated when Cu^2+^ was added

Accession	Description	emPAI				emPAI value ratio (Cu to CK)	
		CK1	Cu1	CK2	Cu2	The first batch	The second batch
C3ER38	Uncharacterized protein	ND	0.18	ND	0.18	–	–
M4LAX0	Uncharacterized protein	0.10	0.47	ND	0.47	4.65	–
C3CHP5	Small acid-soluble spore protein C5	0.47	0.78	ND	0.47	1.66	–
C3BZ34	3-Oxoacyl-[acyl-carrier-protein] synthase 2	0.11	0.15	ND	0.15	1.36	–
C3CRL9	Glyceraldehyde-3-phosphate dehydrogenase	0.24	0.33	0.04	0.33	1.38	9.10
C3I208	Spore coat protein w	0.10	0.32	0.10	0.32	3.30	3.30
M4L1B2	30S ribosomal protein S11	0.23	0.32	0.15	0.42	1.38	2.79
C3CM51	Glutamine synthetase	0.20	0.29	0.08	0.17	1.49	2.08
C3BX70	Mature parasite-infected erythrocyte surface antigen	0.06	0.10	0.06	0.13	1.52	2.06
A0RHK1	GTP-sensing transcriptional pleiotropic repressor CodY	0.16	0.22	0.05	0.10	1.37	2.05
C3CMC5	Stage V sporulation protein S	0.54	1.37	0.78	1.37	2.54	1.76
D5TTP0	PhaR protein	0.15	0.20	0.15	0.26	1.37	1.75
D5TJY1	Citrate synthase	0.13	0.21	0.10	0.17	1.55	1.72
A0RKS3	Enolase	0.09	0.16	0.09	0.16	1.72	1.72
D5TLE4	Homogentisate 1,2-dioxygenase	0.18	0.25	0.09	0.15	1.37	1.72
C3E6D6	Aldehyde dehydrogenase	0.65	0.92	0.38	0.65	1.41	1.69
C3BYJ8	Uncharacterized protein	0.10	0.33	0.21	0.33	3.31	1.58
C3CPV6	Short chain enoyl-CoA hydratase	0.20	0.44	0.20	0.31	2.20	1.57
C3CDB7	Alkyl hydroperoxide reductase subunit C	0.30	0.43	0.19	0.30	1.40	1.57
K0FJZ8	DNA-binding protein HU	0.27	0.37	0.17	0.27	1.39	1.56
C3E7X2	3-hydroxybutyrate dehydrogenase (BDH)	0.17	0.43	0.11	0.17	2.60	1.54
C3CMB9	Spore coat protein E	0.43	0.91	0.65	0.91	2.10	1.39
Q631K2	ATP-dependent Clp protease proteolytic subunit 2	0.29	0.38	0.21	0.29	1.29	1.38
F0PR22	Elongation factor G	0.11	0.14	0.05	0.06	1.31	1.34
C3ETX6	30S ribosomal protein S6	0.27	0.37	0.37	0.49	1.39	1.30
C3EKF4	Methylmalonate semialdehyde dehydrogenase [acylating]	0.26	0.35	0.20	0.26	1.35	1.28
K0FMV8	Butyryl-CoA dehydrogenase	0.05	0.20	0.11	0.14	3.74	1.27

### qRT-PCR analysis of select transcripts

As previously discussed, Cu^2+^ caused ICP production to increase by 21 %, and proteomic analysis revealed that the ICP are mainly composed of Cry1Ca, Cry1Ac, and Cry1Da. To investigate the effects of Cu^2+^ on ICP expression further, the transcripts of these three crystal proteins were analyzed by qRT-PCR. To determine whether or not changes in the proteome correlate with differences at the mRNA level, the transcripts of nine selected genes were analyzed by qRT-PCR, including five upregulated proteins (PhaR, BDH, EF-G, KAS II, and ALDH), three downregulated proteins (SHDA, ATPSβ, and PrkA), and three proteins showing no difference in proteomes [inosine-5′-monophosphate dehydrogenase (IMPDH), small, acid-soluble spore protein B (SASPB), and ornithine aminotransferase (OAT)].

The growth curves obtained show that after 28 h of cultivation, higher bacterial concentrations were found in Cu than in CK. The pH variation curves show that at 30–36 h of cultivation, the pH of the fermentation broth is higher in Cu than in CK (Fig. 5). Thus, we decided to extract the total RNA of the samples for qRT-PCR analysis at the time point of 31 h.

Strain genome sequencing was limited to frame diagrams. The purpose of sequencing was performed to determine important functional genes than can help design gene-specific primer sequences. qRT-PCR results (Fig. 3) demonstrated that Cry1 Da and Cry1Ca are significantly upregulated at the transcriptional level, which confirms that appropriate amounts of Cu^2+^ have positive effect on 130 kDa ICP (Cry1Da and Cry1Ca) production. qRT-PCR showed positive correlations between changes at the translational and transcriptional levels of PhaR, BDH, EF-G, KAS II, ALDH, IMPDH, SASPB, and OAT but no correlation between changes at these levels of SHDA, ATPSβ, and PrkA.

The protein samples for 2D-LC–MS/MS analysis were extracted after 44 h after cultivation, during which cells are in the spore-release phase. In this period, specific metabolic-related proteins, such as SHDA, ATPSβ, and PrkA, have low transcriptional and translational levels but high degradation rates. As Cu^2+^ shortens the fermentation cycle and releases spores and crystals earlier (Fig. 6), thereby resulting in downregulation phenomena. RNA samples for qRT-PCR analysis were extracted after 31 h of cultivation, during which cells are in the spore-formation phase. In this period, ICP and metabolic-related proteins exhibit high transcriptional and translational levels as well as relatively low degradation rates. These differences may result in the noncorrelation of downregulated proteins with changes at the translational and transcriptional levels. In addition, post-transcriptional regulation may also explain the noncorrelation observed.

### Mechanism of Cu^2+^ in improving crystal protein production

Cu^2+^ brought about changes in fermentation parameters: a prolonged stationary phase, rapid pH rebound, a prolonged pH plateau phase, and lower pH in the plateau phase (Fig. 5). We propose that such differences are related to ICP upregulation. Prolonging the stationary phase may directly bring about increased time for ICP synthesis. pH affects nutrient ionization in the medium, influences nutrient absorption, conversion and utility of nutrients by regulating the activity of various enzymes. Thus, pH finally influences growth, sporulation, and ICP synthesis. If pH does not rebound after the logarithmic phase, *B. thuringiensis* would be unable to form spores and crystals [13]. The present study also showed that the initial pH of the medium influences ICP biosynthesis significantly (Fig. 2a, d).

Upregulation of two PHB metabolism-related proteins, PhaR and BDH (Table 2), is an interesting phenomenon. PHB, a member of the polyhydroxyalkanoate family, is synthesized and accumulated as stored intracellular compounds in the form of insoluble cytoplasmic granules under conditions of nutrient imbalance in several bacterial genera [14–16]. PHB was initially synthesized in the logarithmic phase and gradually degraded and utilized during sporulation in *B. thuringiensis* [17–19]. Navarro et al. [20] reported that a linear relationship exists between δ-endotoxin production and PHB accumulation, and a minimum PHB accumulation of 0.52 mg/L is required before the onset of δ-endotoxin production in *B. thuringiensis* subsp. *kurstaki* HD-73. In other words, PHB synthesis and degradation play critical roles in the highly efficient expression of ICP.

BDH is a key enzyme in the reuse of PHB deposits. PHB degradation is initiated by the action of PHB depolymerase to release the monomer 3-hydroxybutyrate (3HB) [21–23]. Upregulation of BDH indicates increased reuse of PHB deposits in the stationary and decline phases, which provide more substances and energy for ICP synthesis. Both PHB accumulation and growth rate show wild-type levels during growth on ethylamine compounds. These results demonstrate that PhaR controls the acetyl-CoA flux to PHB in this methylotrophic bacterium [24].

Results of the present study show that Cu^2+^ caused upregulation of PhaR and BDH (Fig. 3), and brought about downregulation of environmental information-processing proteins, glycan biosynthesis, metabolism proteins (Fig. 8) and acceleration of cell lysis (Fig. 6). These changes are consistent with those caused by eliminating Cu^2+^ from the medium (Fig. 5). Therefore, we suggest that a prolonged stationary phase and rapid pH rebound are caused by increases in PHB.

emPAI semiquantitative (Additional file 1: Table S3) comparative analysis and qRT-PCR results showed upregulation of EF-G, KAS II, and ALDH in the medium to which Cu^2+^ had been added (Table 2; Fig. 3). EF-G is a translation elongation factor that catalyzes the GTP-dependent ribosomal translocation step during translation elongation [25]. Crystal proteins are proteins produced ribosomally, whose expressions require translation elongation factors. Thus, upregulation of EF-G indicates increased active translation promoting crystal protein production. We suggest that the extra energy required for translation comes from utilization of more PHB deposits.

KAS II takes part in fatty acid synthesis. It catalyzes the condensation reaction of fatty acid synthesis by addition of two carbons from malonyl-ACP to an acyl acceptor and has a preference for short chain acid substrates [26, 27]. ALDH acts on the aldehyde or oxo group of donors with NAD or NADP as an acceptor. It eliminates aldehydes produced by over-oxidation of substances in vivo (such as unsaturated fatty acids) and thus performs an important function in detoxification [28–30]. During alcohol detoxification, ALDH further oxidizes acetaldehyde to acetic acid, which is used in the TCA cycle [31]. Upregulation of KAS II and ALDH support the hypothesis that Cu^2+^ promotes changes in metabolism in *B. thuringiensis* X022.

## Conclusions

**Fig. 9:**
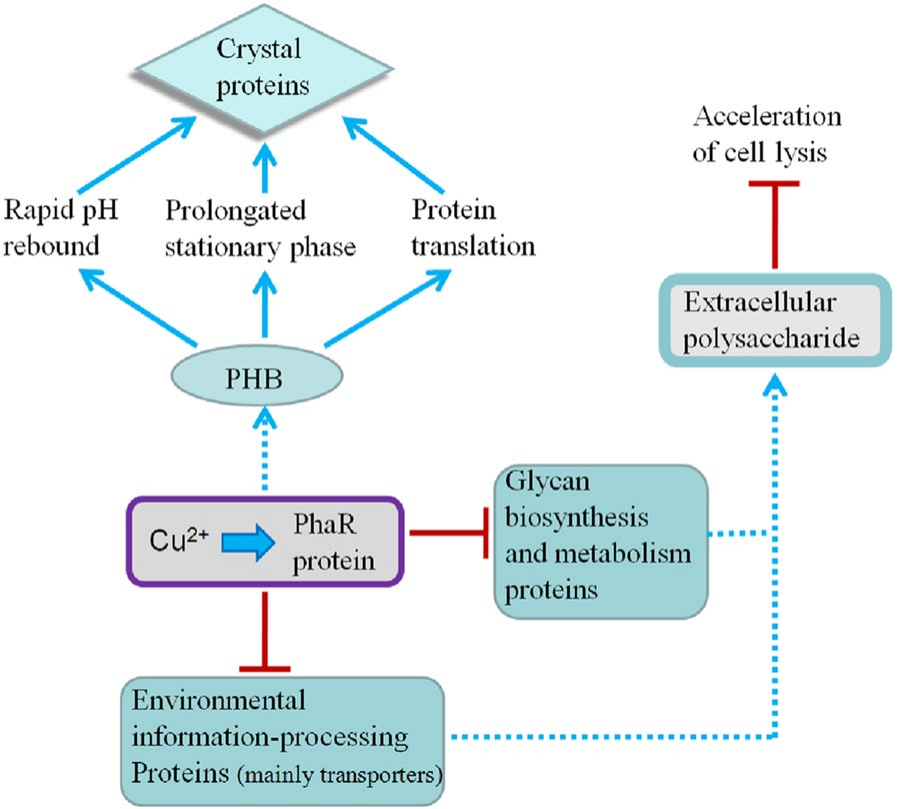
Metabolic changes caused by Cu^2+^ in *B. thuringiensis* X022.  indicates promoting;  indicates promoting speculated;  indicates inhibiting. Cu^2+^ upregulates the expression of PhaR and consequently changes the carbon flow in *B. thuringiensis* X022. Cu^2+^ addition causes downregulation of environmental information-processing proteins, glycan biosynthesis and metabolism proteins, and, therefore, reduces extracellular polysaccharide production, resulting in accelerated cell lysis. More carbon sources can be used to synthesize intracellular PHB. Increases in PHB as a storage material induces a prolonged stationary phase, rapid pH rebound, and releasing more energy for protein translation, ultimately increasing ICP production

In summary, *B. thuringiensis* X022 mainly expresses Cry1Ca, Cry1Ac, and Cry1Da. Cu^2+^ increases the expression of Cry1Da and Cry1Ca, and also enhances the toxicity of fermentation broth to *S. exigua* and *H. armigera*. In this work, mass spectrometry-based proteomics techniques, with qRT-PCR, were used to explore the molecular mechanisms of the effects caused by Cu^2+^ for the first time. Results showed that Cu^2+^ addition causes downregulation of environmental information-processing proteins, glycan biosynthesis and metabolism proteins, and also causes upregulation of PhaR, BDH, EF-G, KAS II, and ALDH. It is suggested that Cu^2+^ increased the expression of PhaR and consequently changed the carbon carbon-energy flow. Thereby reducing extracellular polysaccharide production and accelerating cell lysis. More carbon sources can be used to synthesize intracellular PHB. Increases in PHB as a storage material induces a prolonged stationary phase, rapid pH rebound, and releasing more energy for protein translation, ultimately raising ICP production (Fig. 9).

## Methods

### 16S rRNA gene sequencing and analysis

The novel wild-type strain *B. thuringiensis* X022 (CCTCC No. M2014158) was isolated from soil of China. Total DNA extraction procedures was carried out using E.Z.N.A.^®^ Bacterial DNA Kit (Omega Bio-Tek, America). 16S rRNA gene was amplified from the extracted total DNA with universal primers (Additional file 1: Table S1) and purified with DNA Purification Kit (BioTeke Corporation, Beijing, China). The purified 16S rRNA DNA fragment was ligated to pMD18-T Vector with T4 DNA ligase (TaKaRa, Japan). The product of ligation was transfected into *Escherichia coli* DH5α (stored in our laboratory). Then the right transformant containing pMD18-T-BtX022-16S was screened and cultivated for plasmid extraction and sent to Invitrogen Corporation. The 16S rRNA sequence of *B. thuringiensis* X022 was deposited in the GenBank database (Accession No. KJ698649).

### Detecting effects of pH and Cu^2+^ on ICP biosynthesis

*B. thuringiensis* X022 was transferred from LB liquid medium to fermentation medium with 1 % inoculum. The original fermentation medium contained 18 g/L glucose, 14.5 g/L tryptone, 2.5 g/L K_2_HPO_4_, 0.02 g/L FeSO_4_·7H_2_O, 0.02 g/L MnSO_4_·H_2_O, 0.25 g/L MgSO_4_·7H_2_O. For different initial pH assay, it was adjusted to different pH values using NaOH solution before sterilization. For the Cu^2+^ assay, the calculated amount of CuSO_4_ solution was added before sterilization. Each kind of medium was carried out in three replicates. The protoxins relative concentrations were determined by SDS-PAGE [7] and the statistical significance of ICP biosynthesis determined by SDS-PAGE using the Gro-gel biomedical image analysis software MiBio Statistical significance was evaluated using one-factor and repeated-measures analysis of variance via the general linear statistical model in SPSS. The data reported represent three independent experiments.

### Bioassays of insecticidal activity

*B. thuringiensis* X022 was grown in LB (fermentation broth culture) overnight. The toxicity of spore-crystal mixtures was determined using fivefold dilutions ranging from 0.5 to 100 μL/mL, and these mixtures were added to the artificial diet. Each group contained 24 × 3 larvae, which were reared on an artificial diet. The plate was sealed and placed in a humidified growth chamber at 28 ± 1 °C. With a photoperiod of 16:8 light:dark. Each dose was used in triplicate to establish the toxin potency. The toxicity of spore–crystal mixtures was determined using fivefold dilutions ranging from 0.5 to 100 μl/mL. Each dilution was tested against the first instar, *S. exigua* and *H. armigera* and by oral administration thrice as previously reported [32]. The experiments were repeated three times. After 48 h, the mortality was recorded, and 50 % lethal concentrations (LC_50_) were determined by probit analysis using SPSS software. Non-overlapping 95 % confidence intervals of LC_50_ were used as the criteria to determine significant difference of toxicities among different formulations of acetamiprid [33].

### Cu^2+^ concentration and growth parameters analysis

It was transferred into original fermentation medium (pH 8.0, CK) and 10–6 mol/L Cu^2+^ added fermentation medium (pH 8.0, Cu) respectively. In the lag phase (0–4 h), logarithmic phase (4–18 h), and early stage of the stationary phase (18–28 h), samples were taken at intervals of every 2 h, and after proper dilution (diluted 30-fold in this study), calculated cell using a hemocytometer analysis, under a microscope, pH measurements. Glucose concentration in medium was measured using 3,5-dinitrosalicylic acid method as Yang et al. previously described [34]. After 28 h of cultivation, *B. thuringiensis* X022 in CK but remained in the stationary phase for another 8 h in Cu were observed from this time onward.

### Total bacterial proteins extraction, trypsin digestion

*B. thuringiensis* X022 cultivated in original fermentation medium and 10^−6^ mol/L Cu^2+^ added fermentation medium were harvested from three replicates and mixed together at the time point of 44 h cultivation. The procedure of proteins extraction and trypsin digestion was carried out as previously described [34, 35]. Briefly, after washed with PBS buffer (10 mM, pH 7.8), The cells were mechanically disrupted with disposable grinding pestles, and then treated with ultrasonication (SCIENTZ 98-III) for 10 min at 4 °C. After incubated at 4 °C for 30 min, the mixtures were centrifugation with speed of 12,000*g* at 4 °C for 30 min. The supernatant of proteins was quantified using 2D Quant kit (Amersham Biosciences, Piscataway, NJ, USA). The proteins extracted for 2D-LC–MS/MS analysis were tested with SDS-PAGE (Additional file 1: Fig. S1A). Then they were further digested by trypsin. Firstly they were reduced by 5 mM dithiothreitol (DTT) at 37 °C for 60 min and then were alkylated by 15 mM iodoacetamide at room temperature in the dark for 60 min. The excess iodoacetamide was quenched by adding 15–20 mM DTT and incubating at room temperature for 15 min. After diluting the urea concentration to 1 mol/L using NH_4_HCO_3_ solution, the samples were then incubated with trypsin in a trypsin/protein ratio of 1:50 (w/w) at 37 °C overnight. They were further desalted and concentrated using an Oasis HLB sample cartridge column (Waters Corporation). Finally, the purified peptides were dried at 4 °C by vacuum freeze-drying. Two independent samples were prepared and processed (biological replicates) for each test.

### 2D-LC–MS/MS analysis

The four trypsin digested samples (two replicates) were separated by 2D-HPLC equipped with a strong cation-exchange column (BioBasic SCX; 0.32 mm × 100 mm, 5 μm) and a reversed-phase column (BioBasic-C18; 0.1 mm × 150 mm, 5 μm), and then analyzed by MS/MS using an LTQ XL mass spectrometer (ThermoFisher, San Jose, CA, USA) equipped with a homemade nano-ionization source. The procedures were carried out as Huang et al. clearly described previously [10].

### Database search and functional classification

An in-house database was constructed with the protein sequences downloaded from the Uniprot Knowledgebase (Swiss-Prot plus TrEMBL) protein database (http://www.uniprot.org) as a FASTA-formatted sequence that included all *B. thuringiensis* subspecies. And the search results were further validated manually and classified into functional categories according to their annotated functions in the Uniprot Knowledgebase as well as to homology/functions according to the BioCyc (http://www.biocyc.org/) and KEGG (http://www.genome.ad.jp/kegg/kegg2.html) metabolic pathway databases [11].

### Semiquantification analysis of proteins expression

Protein abundance was determined by semiquantitative analysis as described earlier [36, 37]. Briefly, each protein abundance was obtained from emPAI value. The emPAI semi-quantitative of ICPs, in Additional file 1: Table S3, which calculated as emPAI = 10^SC/OP^ − 1. SC is the total spectral count of MS/MS spectra for each detected protein. The OP was obtained after in silico trypsinization of the protein by using the IPEP online proteolysis (http://ipep.moffitt.org/searchProtein.cgi) with parameters of 600–3500 Da mass spectrometer and two maximum missed cleavages. Protein abundance was considered significantly up- or down-regulated when the emPAI ratios between two medium from two replicates were both higher than 1.25 or lower than 0.8, respectively.

### Quantitative real-time RT-PCR

The relative mRNA levels of selected genes were measured by a two-step real-time RT-PCR analysis with an ABI 7500 Real-Time PCR System (Applied Biosystems, USA) using Power SYBR^®^ Green PCR Master Mix (Applied Biosystems) as previously described [37]. The sequences of the primers used in real-time PCR were developed with Primer version 5.00 (Premier Biosoft International, Palo Alto, CA, USA) and gene-specific primers sequences (Additional file 1: Table S1). For qRT-PCR analysis, bacteria were harvested at the time point of 31 h cultivation. The procedures were carried out as described previously. Briefly, the total RNA was isolated using TRIzol Reagent (Invitrogen). The quality and the integrity of the RNA samples were evaluated by absorbance measurements (Thermo Scientific NanoDrop 2000 Spectrophotometers) and agarose electrophoresis (Additional file 1: Fig. S2). Genomic DNA was removed from the total RNA using DNase I (Fermentas) and then the total RNA was retrotranscribed to cDNA using RevertAid™ First Strand cDNA Synthesis Kit (Fermentas) according to the manufacturer’s procedure. The cDNAs were used as templates to perform relative quantitative real-time PCR with 16S rRNA as endogenous control. The relative quantification method (delta–delta threshold cycle) was used to evaluate quantitative variation between samples examined. mRNA abundance was considered significantly up- or down-regulated when the ratios between two medium were higher than 1.5 or lower than 0.75, respectively.

## Additional files

10.1186/s12934-015-0339-9 SDS-PAGE of whole proteins extracted from *B. thuringiensis* strain. **Figure S2.** Integrity detection of the RNA samples extracted. **Table S1.** Primers for quantitative RT-PCR analysis and 16S rRNA gene sequencing. **Table S2.** The list of identified proteins and their internal tryptic peptides from strain *B. thuringiensis* X022. **Table S3.** The emPAI semi-quantitative of ICPs. **Table S4.** The list of the proteins down-regulated when Cu^2+^ was added.
10.1186/s12934-015-0339-9 Identification of B. thuringiensis proteins without and with Cu^2+^ added in the medium and biological replicates.
10.1186/s12934-015-0339-9 Functional classification of proteins identified from strain X022 without Cu^2+^ added in the medium.
10.1186/s12934-015-0339-9 Functional classification of proteins identified from strain X022 with Cu^2+^ added in themedium.

## Abbreviations

qRT-PCR: quantitative RT-PCR
2D-LC–MS/MS: two-dimensional liquid chromatography–tandem mass spectrometry
ICP: insecticidal crystal proteins
PHB: poly-β-hydroxybutyrate
KAS II: 3-oxoacyl-[acyl-carrier-protein] synthase 2
ALDH: aldehyde dehydrogenase
EF-G: elongation factor G
BDH: 3-hydroxybutyrate dehydrogenase
SHDA: succinate dehydrogenase subunit A
ATPSβ: ATP synthase subunit beta
IMPDH: inosine-5′-monophosphate dehydrogenase
SASPB: small, acid-soluble spore protein B
OAT: ornithine aminotransferase
3HB: 3-hydroxybutyrate
