# Supplementary material for: Proteomic analysis of the influence of Cu2+ on the crystal protein production of Bacillus thuringiensis X022
Source: Microb Cell Fact. 2015 Oct 5;14:153. doi: 10.1186/s12934-015-0339-9 (PMC4595308; doi:10.1186/s12934-015-0339-9)
Supplement: Supplementary file 1 — 10.1186/s12934-015-0339-9 SDS-PAGE of whole proteins extracted from B. thuringiensis strain. Figure S2. Integrity detection of the RNA samples extracted. Table S1. Primers for quantitative RT-PCR analysis and 16S rRNA gene sequencing. Table S2. The list of identified proteins and their internal tryptic peptides from strain B. thuringiensis X022. Table S3. The emPAI semi-quantitative of ICPs. Table S4. The list of the proteins down-regulated when Cu2+ was added. [file 12934_2015_339_MOESM1_ESM.doc]

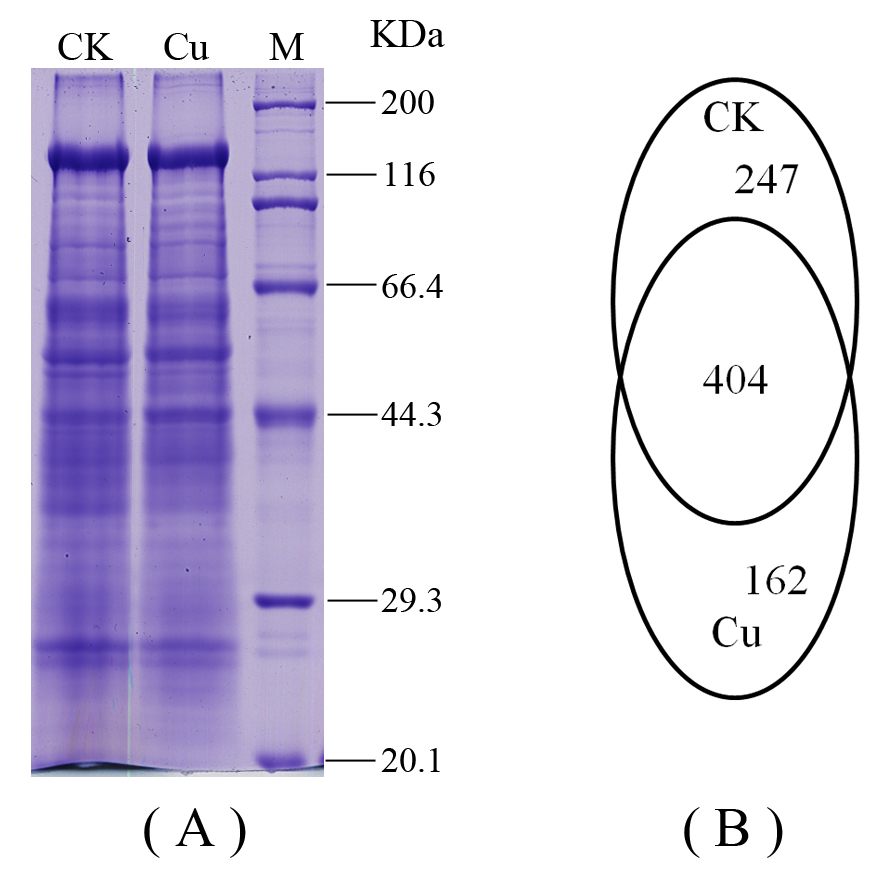


Figure S1. SDS-PAGE of whole proteins extracted from *B. thuringiensis* strain (A) and distribution of the whole cell proteins of *B. thuringiensis* strain cultured in two medium (identified by 2D-LC-MS/MS) (B). CK: proteins from the original medium; Cu: proteins from the 10^-6^ mol/L Cu^2+^ added medium.


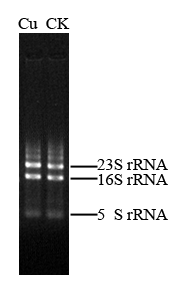


Figure S2. Integrity detection of the RNA samples extracted. Agarose gel electrophoresis is performed. Cu: RNA extracted from the original medium, OD_260_ / OD_280_ ＝ 2.04; CK: RNA extracted from the Cu^2+^ added medium, OD_260_ / OD_280_＝2.05.

Table S1

Primers for quantitative RT-PCR analysis and 16S rRNA gene sequencing

| **Gen** | **Primer name** | **Primer Sequence** | **production size** |
| --- | --- | --- | --- |
| 16S rRNA-1 | 16S1-F | CTTGACATCCTCTGAAAACCCTA | 107 |
|  | 16S1-R | GACTTAACCCAACATCTCACGAC |  |
| Cry1Ca | Cry1Ca-F | GGATTGGTTTAGTGTTGGACGC | 197 |
|  | Cry1Ca-R | GCCAAGGTTGCTGTAATAATCGTA |  |
| Cry1Da | Cry1Da-F | CTGGAATCGAGGGAGTGGAA | 109 |
|  | Cry1Da-R | GATACGCTGGCATCTTGAGG |  |
| Cry1Ac | Cry1Ac-F | TGTTTCAATGTTTCGTTCAGGC | 87 |
|  | Cry1Ac-R | TCAGCACTACGATGTATCCAAGAG |  |
| EF-G | EF-G-F | AAAACAAGATCGTCGGTGGTG | 142 |
|  | EF-G-R | TGGTAAGATCCGTCAACTAATGC |  |
| KASⅡ | KASⅡ-F | GTAGCGGTTTTGTAATGGGTGA | 142 |
|  | KASⅡ-R | GCAGGCATTGTAATATGGAACG |  |
| ALDH | ALDH-F | TCCTTGGTCTCGTATGAGCACTG | 117 |
|  | ALDH-R | GGCTTTCCGTTATCTAATGTTTCG |  |
| SHDA | SHDA-F | CCAACCACCAGTTAAAGCAATG | 119 |
|  | SHDA-R | CACCAAATCGACGGAAATCA |  |
| ATPSβ | ATPSβ-F | TGGTGTAGGTGAGCGTACTCGT | 110 |
|  | ATPSβ-R | CAGGTGGCTCGTTCATTTGTC |  |
| PrkA | PrkA-F | ATTGGTACATTTAGCCCGTCTG | 157 |
|  | PrkA-R | CCATCATTCCTCGGTTTGC |  |
| IMPDH | IMPDH-F | GTGTATCGTGGTATGGGTTCTGT | 101 |
|  | IMPDH-R | CCTTCAATACCTTCTGGGACAA |  |
| SASPB | SASPB-F | ATCGCTCAAGAGTTTGGTGTTC | 118 |
|  | SASPB-R | AACCGCCTAATTGTTGCTCAG |  |
| OAT | OAT-F | GCTTCCTGGCATCGTTGTAA | 105 |
|  | OAT-R | GCTTCACCTTGAATTGGCTCTA |  |
| 16S rRNA-for sequencing | BF | AGAGTTTGATCCTGGCTCAG | 1515 |
|  | BR | ACGGCTACCTTGTTACGACTT |  |

# Table S2

# The list of identified proteins and their internal tryptic peptides from strain *B. thuringiensis* X022

| **Accession No** | | | **Protein name** | **MW(kDa)** | | | **pI** | **Score(Cu/CK)** | | | **Unique Peptides** | |
| --- | --- | --- | --- | --- | --- | --- | --- | --- | --- | --- | --- | --- |
|  | **Precursor_Mass** | | | | **Xcorr** | **Charge** | | **Missed Cleavages** | | | |  |
| **UniprotKB:** | | **Cry1Ca** | | | **134.7** | | **5.07** | **582/476** | |  | | |
| P0A376 | | 2821.90 | | | 6.75 | | 3 | 1 | NFYWGGHRVISSLIGGGNITSPIYGR | | | |
| CK:40.96%Cu:46.76%Sequence Coverage(average from two replicats) | | 2864.75 | | | 6.73 | | 3 | 1 | YNAKHEIVNVPGTGSLWPLSAQSPIGK | | | |
|  |  | 3549.69 | | | 5.79 | | 3 | 1 | LLQQPWPAPPFNLRGVEGVEFSTPTNSFTYR | | | |
|  |  | 2506.32 | | | 5.67 | | 3 | 2 | GFRVWGGTSVITGPGFTGGDILRR | | | |
|  |  | 1785.44 | | | 4.77 | | 3 | 1 | TFRYTDFSNPFSFR | | | |
|  |  | 2250.36 | | | 4.69 | | 2 | 0 | NTFGDFVSLQVNINSPITQR | | | |
|  |  | 1891.75 | | | 4.38 | | 2 | 0 | GVEGVEFSTPTNSFTYR | | | |
|  |  | 2260.82 | | | 4.31 | | 3 | 1 | LIRHIDEYADHcANTYNR | | | |
|  |  | 2199.40 | | | 4.17 | | 2 | 0 | DLTLTVLDIAAFFPNYDNR | | | |
|  |  | 2588.79 | | | 4.13 | | 3 | 1 | ENPcESNRGYGDYTPLPAGYVTK | | | |
|  |  | 1805.07 | | | 4.12 | | 2 | 0 | VISSLIGGGNITSPIYGR | | | |
|  |  | 1893.02 | | | 4.06 | | 2 | 0 | SGTPFLTTGVVFSWTHR | | | |
|  |  | 2388.06 | | | 4.00 | | 3 | 0 | HEIVNVPGTGSLWPLSAQSPIGK | | | |
|  |  | 1380.62 | | | 3.89 | | 2 | 0 | YTDFSNPFSFR | | | |
|  |  | 3822.24 | | | 3.86 | | 3 | 1 | NAAIANLEGLGNNFNIYVEAFKEWEEDPNNPATR | | | |
|  |  | 2354.45 | | | 3.58 | | 3 | 1 | DLTLTVLDIAAFFPNYDNRR | | | |
|  |  | 2105.34 | | | 3.58 | | 2 | 1 | NVIKNGDFNNGLLcWNVK | | | |
|  |  | 1250.67 | | | 3.29 | | 2 | 0 | TMEIGENLTSR | | | |
|  |  | 1677.07 | | | 3.27 | | 2 | 0 | LLQQPWPAPPFNLR | | | |
|  |  | 2596.13 | | | 3.03 | | 3 | 0 | ISGFEVPLLSVYAQAANLHLAILR | | | |
|  |  | 3608.15 | | | 2.90 | | 3 | 2 | DLTLTVLDIAAFFPNYDNRRYPIQPVGQLTR | | | |
|  |  | 2327.18 | | | 2.78 | | 2 | 0 | VIVLTGAASTGVGGQVSVNMPLQK | | | |
|  |  | 1879.61 | | | 2.78 | | 3 | 0 | HIDEYADHcANTYNR | | | |
|  |  | 2147.07 | | | 2.59 | | 2 | 1 | VWGGTSVITGPGFTGGDILRR | | | |
|  |  | 1427.92 | | | 2.56 | | 2 | 1 | RYPIQPVGQLTR | | | |
| **UniprotKB:** | | **Cry1Ac** | | | **133.2** | | **5.14** | **568/466** | |  | | |
| E9K6H7 CK:41.71%Cu:40.96%SequenceCoverage(average from two replicats) | | 2852.12 | | | 6.24 | | 3 | 1 | YNAKHETVNVPGTGSLWPLSAQSPIGK | | | |
|  |  | 3373.43 | | | 6.04 | | 3 | 1 | NFSGTAGVIIDRFEFIPVTATLEAEYNLER | | | |
|  |  | 2211.44 | | | 5.49 | | 2 | 0 | GNFLFNGSVISGPGFTGGDLVR | | | |
|  |  | 2554.26 | | | 5.27 | | 3 | 1 | SIRSPHLMDILNSITIYTDAHR | | | |
|  |  | 3729.44 | | | 5.07 | | 3 | 0 | RPFNIGINNQQLSVLDGTEFAYGTSSNLPSAVYR | | | |
|  |  | 3700.81 | | | 4.69 | | 3 | 2 | NFSGTAGVIIDRFEFIPVTATLEAEYNLERAQK | | | |
|  |  | 2117.56 | | | 4.62 | | 2 | 0 | SAEFNNIIASDSITQIPAVK | | | |
|  |  | 2376.00 | | | 4.16 | | 3 | 0 | HETVNVPGTGSLWPLSAQSPIGK | | | |
|  |  | 1728.77 | | | 4.15 | | 2 | 1 | GYRDYTPLPVGYVTK | | | |
|  |  | 2359.32 | | | 4.07 | | 3 | 1 | NRGYNEAPSVPADYASVYEEK | | | |
|  |  | 2699.93 | | | 3.95 | | 3 | 1 | EIYTNPVLENFDGSFRGSAQGIER | | | |
|  |  | 1705.18 | | | 3.35 | | 2 | 0 | GYIEVPIHFPSTSTR | | | |
|  |  | 1284.97 | | | 3.27 | | 2 | 0 | LIGNYTDYAVR | | | |
|  |  | 1308.75 | | | 3.10 | | 2 | 2 | IDESKLKAFTR | | | |
|  |  | 2864.56 | | | 2.85 | | 3 | 2 | ELTLTVLDIVALFPNYDSRRYPIR | | | |
|  |  | 2088.25 | | | 2.67 | | 3 | 2 | NLLQDSNFKDINRQPER | | | |
|  |  | 1490.77 | | | 2.67 | | 3 | 2 | RYPIRTVSQLTR | | | |
|  |  | 2305.32 | | | 2.87 | | 3 | 1 | LIGNYTDYAVRWYNTGLER | | | |
|  |  | 1902.21 | | | 2.52 | | 3 | 2 | SYTDGRRENPcEFNR | | | |
| **UniprotKB:** | | **Cry1Da** | | | **132.4** | | **5.25** | **370/295** | | | | |
| P19415 | | 2715.14 | | | 5.90 | | 3 | 0 | SGPIDSFSELPPQDASVSPAIGYSHR | | | |
| CK:27.27%Cu:30.85%SequenceCoverage (average from two replicates) | | 2870.25 | | | 5.54 | | 3 | 0 | YSDLTSLIHVYTNHcVDTYNQGLR | | | |
|  |  | 2327.39 | | | 4.56 | | 3 | 1 | AHTLASGASVIKGPGFTGGDILTR | | | |
|  |  | 2297.57 | | | 4.43 | | 2 | 0 | SPHLVDFLNSFTIYTDSLAR | | | |
|  |  | 1726.17 | | | 4.38 | | 2 | 0 | SFAHTTLFTPITFSR | | | |
|  |  | 2809.01 | | | 4.26 | | 3 | 1 | NRGYDEAYGNNPSVPADYASVYEEK | | | |
|  |  | 2845.47 | | | 4.07 | | 3 | 1 | SPLYGREGNTERPVTITASPSVPIFR | | | |
|  |  | 1570.68 | | | 3.62 | | 2 | 0 | YAYWGGHLVNSFR | | | |
|  |  | 2171.86 | | | 3.35 | | 3 | 0 | EGNTERPVTITASPSVPIFR | | | |
|  |  | 1275.03 | | | 3.33 | | 2 | 0 | IAGTVFSWTHR | | | |
|  |  | 1312.44 | | | 3.24 | | 2 | 0 | FLSDWIVYNR | | | |
|  |  | 1701.57 | | | 3.00 | | 2 | 1 | VTFTGRLPQSYYIR | | | |
|  |  | 2527.92 | | | 2.94 | | 3 | 1 | YAYWGGHLVNSFRTGTTTNLIR | | | |
|  |  | 1147.21 | | | 2.80 | | 3 | 0 | LcHATFLER | | | |
|  |  | 2388.07 | | | 2.56 | | 3 | 1 | AFSDWEKDPTNPALREEMR | | | |
| **UniprotKB** | | **Cry1F** | | | **132.1** | | **5.31** | **122/0** |  | | | |
| B2ZPN4 | | 2113.60 | | | 4.46 | | 2 | 0 | LGNLEFLEEEPLLGEALAR | | | |
| Cu: 10.62% Sequence Coverage | | | | | | | | | | | | |
| **UniprotKB** | | **Cry1 type crystal protein** | | | **89.0** | | **6.64** | **4.69/0** | | | | |
| Q6PYW6 | | 2868.44 | | | 4.69 | | 3 | 1 | YNAKHETLNVPGTGSLWPLAAESSIGR | | | |
| Cu: 3.45% Sequence Coverage | | | | | | | | | | | | |
| **UniprotKB** | | **Insecticidal protein 2** | | | **129.9** | | **5.20** | **251/0** | | | | |
| C8CBP2 | | 1973.23 | | | 3.78 | | 2 | 0 | SVLVVLEWEAEVSQEVR | | | |
| Cu: 15.83% Sequence Coverage | | | | | | | | | | | | |

Table S3

The emPAI semi-quantitative of ICPs

| **Accession** | **Description** | **emPAI** | | | | **emPAI value Ratio (Cu to CK)** | |
| --- | --- | --- | --- | --- | --- | --- | --- |
|  |  | **CK_1_** | **Cu_1_** | **CK_2_** | **Cu_2_** | **The first batch** | **The second batch** |
| P0A376 | Cry1Ca | 0.54 | 0.66 | 0.45 | 0.61 | 1.23 | 1.33 |
| P19415 | Cry1Da | 0.21 | 0.25 | 0.21 | 0.30 | 1.20 | 1.47 |
| E9K6H7 | Cry1Ac | 0.53 | 0.55 | 0.44 | 0.58 | 1.04 | 1.33 |

Table S4

The list of the proteins down-regulated when Cu^2+^ was added

| **Accession** | **Description** | **emPAI** | | | | **emPAI value Ratio (Cu to CK)** | |
| --- | --- | --- | --- | --- | --- | --- | --- |
|  |  | **CK_1_** | **Cu_1_** | **CK_2_** | **Cu_2_** | **The first batch** | **The second batch** |
| C3CMC2 | Pyruvate ferredoxin oxidoreductase, beta subunit | 0.17 | 0.06 | 0.11 | ND | 0.32 | ND |
| C3CDS5 | Protein PrkA | 0.14 | 0.08 | 0.15 | 0.04 | 0.57 | 0.29 |
| C3H439 | Extracellular solute-binding protein family 5 | 0.10 | 0.07 | 0.09 | 0.03 | 0.70 | 0.32 |
| C3CNT6 | 2-oxoisovalerate dehydrogenase subunit alpha | 0.11 | 0.08 | 0.08 | 0.03 | 0.74 | 0.32 |
| A0RL96 | ATP synthase gamma chain | 0.18 | 0.11 | 0.18 | 0.07 | 0.58 | 0.38 |
| C3CKZ5 | Uncharacterized protein | 0.35 | 0.11 | 0.57 | 0.22 | 0.30 | 0.39 |
| D5TL40 | Phosphoenolpyruvate carboxykinase [ATP] | 0.12 | 0.06 | 0.18 | 0.09 | 0.49 | 0.48 |
| M4L8P5 | Uncharacterized protein | 0.12 | 0.08 | 0.16 | 0.08 | 0.65 | 0.48 |
| A0RHE6 | Pyruvate ferredoxin oxidoreductase, alpha subunit | 0.07 | 0.04 | 0.11 | 0.05 | 0.49 | 0.49 |
| A0RJC0 | Succinate dehydrogenase subunit A | 0.25 | 0.15 | 0.11 | 0.05 | 0.59 | 0.49 |
| C3CQM3 | ABC transporter substrate-binding protein | 0.10 | 0.06 | 0.06 | 0.03 | 0.66 | 0.49 |
| Q3ERF7 | RNA polymerase sigma factor | 0.05 | 0.03 | 0.05 | 0.03 | 0.49 | 0.49 |
| C3DJX0 | Uncharacterized protein | 0.05 | 0.02 | 0.05 | 0.02 | 0.49 | 0.49 |
| C3CCN6 | Negative regulator of genetic competence | 0.02 | 0.01 | 0.02 | 0.01 | 0.50 | 0.50 |
| Q3EMZ7 | Propionyl-CoA carboxylase beta chain | 0.09 | 0.07 | 0.12 | 0.07 | 0.74 | 0.59 |
| C3CND0 | 5-methyltetrahydropteroyltriglutamate-homocysteine methyltransferase | 0.08 | 0.05 | 0.08 | 0.05 | 0.59 | 0.59 |
| C3CPT3 | Spore germination protein GerE | 0.74 | 0.17 | 0.27 | 0.17 | 0.23 | 0.64 |
| C3DKV0 | N-acetylmuramoyl-L-alanine amidase | 0.18 | 0.07 | 0.10 | 0.07 | 0.38 | 0.66 |
| C3ERH8 | Glyceraldehyde-3-phosphate dehydrogenase | 0.09 | 0.06 | 0.09 | 0.06 | 0.66 | 0.66 |
| Q3EUT8 | S-adenosylmethionine synthase | 0.09 | 0.06 | 0.09 | 0.06 | 0.66 | 0.66 |
| A0R8I6 | 30S ribosomal protein S3 | 0.08 | 0.05 | 0.08 | 0.05 | 0.66 | 0.66 |
| C3DR70 | Isocitrate dehydrogenase [NADP] | 0.10 | 0.05 | 0.08 | 0.05 | 0.49 | 0.66 |
| M4L3X2 | Asparagine synthetase | 0.31 | 0.21 | 0.16 | 0.12 | 0.69 | 0.74 |
| C3ETG1 | ATP synthase subunit beta | 0.67 | 0.42 | 0.46 | 0.35 | 0.63 | 0.75 |
| C3C5N7 | Aconitate hydratase | 0.08 | 0.04 | 0.07 | 0.06 | 0.49 | 0.79 |
